# Novel combinations of PI3K-mTOR inhibitors with dacomitinib or chemotherapy in PTEN-deficient patient-derived tumor xenografts

**DOI:** 10.18632/oncotarget.19109

**Published:** 2017-07-08

**Authors:** Irene Brana, Nhu-An Pham, Lucia Kim, Shingo Sakashita, Ming Li, Christine Ng, Yuhui Wang, Peter Loparco, Rafael Sierra, Lisa Wang, Blaise A. Clarke, Benjamin G. Neel, Lillian L. Siu, Ming-Sound Tsao

**Affiliations:** ^1^ Princess Margaret Cancer Centre, University Health Network, University of Toronto, Toronto, Ontario, Canada; ^2^ Department of Pathology and Laboratory Medicine, University Health Network, University of Toronto, Toronto, ON, Canada

**Keywords:** PI3K, PTEN, xenograft, chemotherapy, dacomitinib

## Abstract

PTEN inactivation occurs commonly in human cancers and putatively activates the PI3K/AKT/ mTOR pathway. Activation of this pathway has been involved in resistance to chemotherapy or anti-EGFR/HER2 therapies. We evaluated the combination of PI3K-mTOR inhibitors with chemotherapy or the pan-HER inhibitor dacomitinib in PTEN-deficient patient-derived tumor xenografts (PDX).

Three PDXs were selected for their lack of PTEN expression by immunohistochemistry: a triple-negative breast cancer (TNBC), a *KRAS* G12R low-grade serous ovarian cancer (LGSOC), and *KRAS* G12C and *TP53* R181P lung adenocarcinoma (LADC). Two dual PI3K-mTOR inhibitors were evaluated—PF-04691502 and PF-05212384—in combination with cisplatin, paclitaxel, or dacomitinib.

The addition of PI3K-mTOR inhibitors to cisplatin or paclitaxel increased the activity of chemotherapy in the TNBC and LGSOC models; whereas no added activity was observed in the LADC model. Pharmacodynamic modulation of pS6 and pAKT was observed in the group treated with PI3K-mTOR inhibitor.

Our research suggests that the addition of a PI3K-mTOR inhibitor may enhance tumor growth inhibition when compared to chemotherapy alone in certain PTEN-deficient PDXs. However, this benefit was absent in the *KRAS* and *TP53* mutant LADC model. The role of PTEN deficiency in the antitumor activity of these combinations should be further investigated in the clinic.

## INTRODUCTION

The phosphoinositide-3-kinase (PI3K)/Akt/mammalian target of rapamycin (mTOR) pathway is commonly activated in cancer by several mechanism including activating mutations of *PIK3CA* or *AKT1* and or loss of phosphatase and tensin homolog (PTEN) [1] . This signaling pathway is critical in the regulation of cell growth, metabolism and survival, angiogenesis, tumor invasion, cell cycle regulation and DNA repair [2, 3]. The activity of different PI3K inhibitors in unselected population in the clinical setting has been limited [1]. Preclinical testing strategies to increase the antitumor activity of these compounds should address effectiveness of combination regimens with chemotherapy or other targeted agents and improvement of patient selection based on biomarkers predictive of an activated PI3K pathway.

Preclinical and clinical evidence supports a role of the PI3K pathway in chemoresistance in different tumor types including ovarian [4], breast [5], and non-small cell lung adenocarcinoma (LADC) [6]. Furthermore, inhibition of the PI3K pathway sensitizes preclinical models to chemotherapy [6-8]. Likewise, resistance to anti-EGFR and anti-HER2 therapies are associated with PI3K pathway activation by *PIK3CA* mutations in *EGFR* mutant LADC [9], and HER2-positive breast cancer [10] [11], or by loss of PTEN [12] or HER3 activation (which activates the PI3K pathway) in breast cancer models [13]. In cell lines, the addition of PI3K inhibitors overcomes resistance to anti-EGFR or anti-HER2 agents [10, 11, 14]. This evidence has supported the evaluation of the PI3K inhibitors in combination with anti-EGFR or anti-HER2 therapies in the clinical setting and several combinations are under investigation.

Patient derived tumor xenografts (PDXs) represent promising pre-clinical models as they seem to recapitulate some of the molecular characteristics of the primary tumor [15] as well as clinical tumor response [16-18]. To improve the clinical activity of the PI3K inhibitors, we tested several therapeutic strategies in three different tumor types using PDX selected for deficient PTEN expression as increased sensitivity to PI3K and mTOR inhibitors has been previously described in cancer cell lines [19]. PTEN-deficient tumors seem to signal preferentially through the PI3K beta isoform (p110β) [20, 21] but it remains unclear whether single inhibition of the beta isoform would be sufficient to induce tumor growth inhibition, or whether dual PI3K alpha and beta isoform inhibition would be superior [22].

In the current study, we evaluated two PI3K-mTOR inhibitors in combination with a pan-HER inhibitor dacomitinib, cisplatin or paclitaxel. The two PI3K-mTOR inhibitors (PF-04691502 and PF-05212384), although different in route of administration and pharmacokinetics, both are potent inhibitors of all PI3K isoforms and mTOR [23, 24] and have shown clinical antitumor activity as monotherapies [25, 26]. Dacomitinib is an irreversible tyrosine kinase inhibitor targeting EGFR, HER2 and HER4 [27] with antitumor activity demonstrated pre-clinically in EGFR wild type and mutant LADC models [28], as well as clinically in head and neck squamous cell carcinoma [29] and LADC [30]. Cisplatin is commonly used as the backbone of chemotherapy regimens for many cancers, including LADC [31], ovarian [32], and triple negative breast cancers (TNBC) [33]. Likewise, paclitaxel is widely used in LADC [31], ovarian [32] , and breast cancers [34]. We hypothesize that in tumors deficient in PTEN protein expression, PI3K inhibition might increase the activity of cisplatin, paclitaxel or dacomitinib. We expect that the simultaneous evaluation of these compounds in PDX may expedite the translation of the most promising combinations into the clinical setting.

## RESULTS

### Molecular characterization

Three PDX models were selected based on their deficient (null or low) PTEN expression. The three models display morphological and molecular characteristics from the original tumor (Supplementary Figure 1). The TNBC model lacks PTEN expression and did not harbor any of the mutations in the Oncocarta panel. PTEN staining was faint by immunohistochemistry in the low-grade serous ovarian cancer (LGSOC) and LADC PDX models. Furthermore, both of the latter models had *KRAS* mutations (G12R and G12C respectively in the LGSOC and LADC models). The LADC model also had a *TP53* co-mutation (R181P) detected by direct sequencing.

### PI3K-mTOR inhibitor and Cisplatin

**Figure 1:**
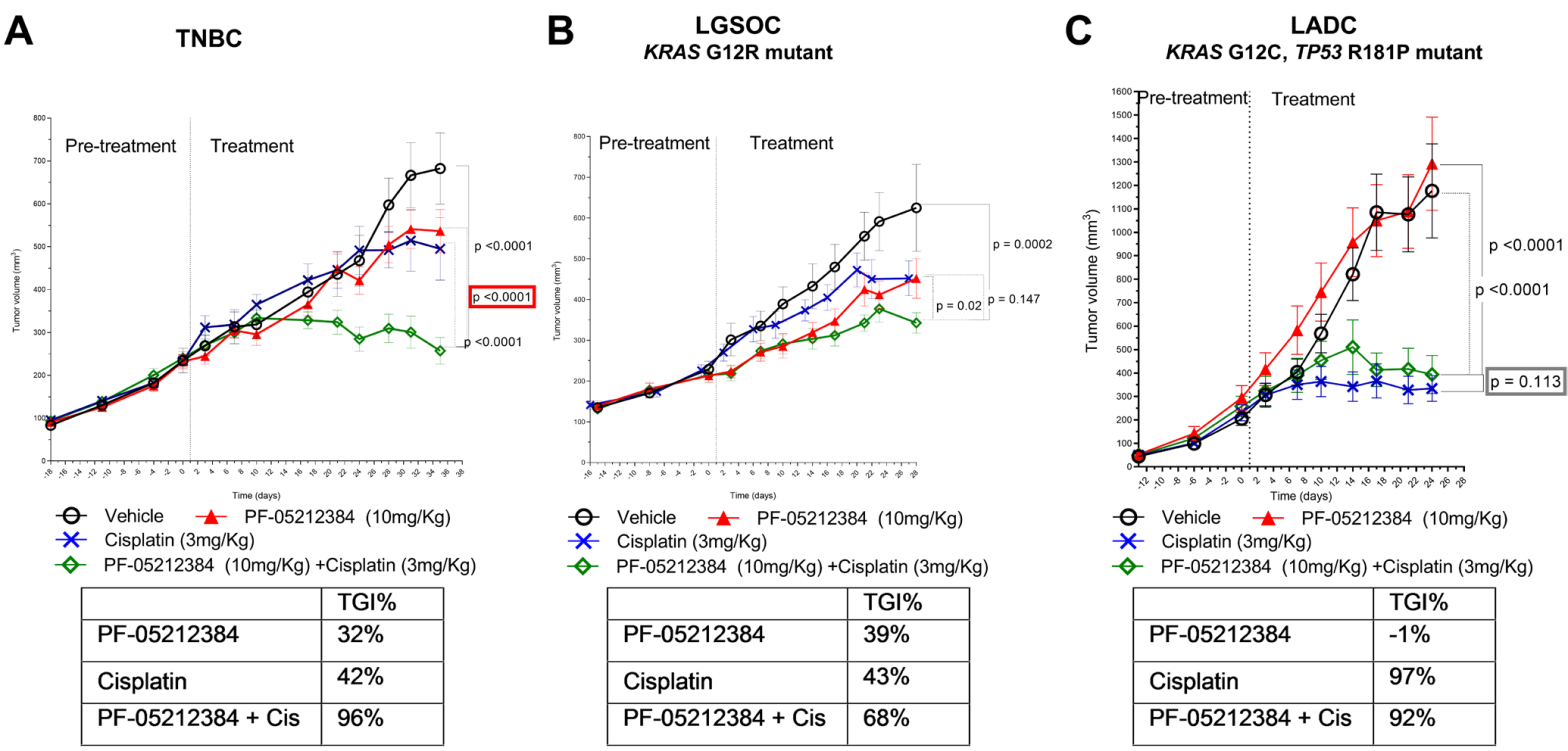
Combined cisplatin and PI3K-mTOR inhibitor in PTEN-deficient gPDX Tumor growth of **A.** triple-negative breast cancer (TNBC), **B.** *KRAS* mutant (G12R) low-grade ovarian cancer (LGSOC) and **C.** *TP53* (R181P) and *KRAS* (G12C) mutant lung adenocarcinoma (LADC). The three models were treated with vehicle, PF-05212384 (10 mg/kg, twice weekly, intravenously, cisplatin (3 mg/kg, once weekly, intraperitoneally) or the combination of both agents. Relative tumor volumes are displayed as mean +/- SE. p: p value for daily tumor volume change for each arm in comparison to vehicle arm. TGI%: percentages of tumor growth inhibition in comparison to vehicle arm.

Treatment duration was up to 29 days when experiments were discontinued due to inaccessible tail veins for IV drug administration. Single agent PF-05212384 had no significant activity as single agent in any of the three models (Table 1 and Figure 1). As a single agent, cisplatin showed insignificant anti-tumor activity in the TNBC (Figure 1A) and LGSOC models (Figure 1B), but induced tumor growth inhibition (TGI) > 50% in the LADC model (Figure 1C). The combination of cisplatin and PF-05212384 induced TGI > 50% in all the three models (Table 1). While the combination was synergistic in the TNBC model (*p* < 0.05), PF-05212384 did not enhance the TGI induced by cisplatin single agent in the LADC model (*p* = 0.113), nor in the LGSOC model (*p* = 0.147).

**Table 1:** Patient derived xenograft molecular profile and tumor growth inhibition per treatment arm

PDX tumor type	TNBC		LGSOC		LADC	
Molecular profile	PTEN null		*KRAS* G12R PTEN low		*KRAS* G12C *TP53* R181P PTEN low	
	TGI%	ΔV *p*-value	TGI%	ΔV *p*-value	TGI%	ΔV *p*-value
Experiment 1						
PF-05212384	32%	0.07	39%	0.19	-1%	0.78
Cisplatin	42%	<0.05**	43%	0.03	97%	<0.05**
PF-05212384 + Cisplatin	96%	<0.05**	68%	<0.05**	92%	<0.05**
Experiment 2						
PF-05212384	22%	0.64	40%	0.21		
PF-04691502					33%	<0.05**
Paclitaxel	84%	<0.05**	38%	0.20	79%	<0.05**
PF-05212384 + Paclitaxel	110%^	<0.05**	56%	<0.05**		
PF-04691502 + Paclitaxel					45%	<0.05**
Experiment 3						
PF-05212384	22%	0.61	40%	0.26		
PF-04691502					33%	<0.05**
Dacomitinib	15%	0.99	8%	0.53	23%	0.35
PF-05212384 + Dacomitinib	55%	0.09	45%	0.47		
PF-04691502 + Dacomitinib					32%	0.055

TNBC = triple negative breast cancer; LGSOC = low grade serous ovarian cancer; LADC = non-small cell lung cancer; TGI%: percentages of tumor growth inhibition; ΔV: differences between daily tumor volume change of each treatment arm and the control arm; ^ Tumor regression; ** statistically significant

The initial dose selected for PF-05212384 (15 mg/kg) was found to be excessively toxic in the first model evaluated (LADC). Several cases of sudden death occurred after the first administration. Hence, a lower dose (10 mg/kg) was administered in subsequent experiments. After this dose reduction, the treatment was well-tolerated in the three models, with mean weight loss ranging 10 - 15% in the combination arm in the TNBC model and in cisplatin-containing arm in the LGSOC model. No mean weight loss was observed in the LADC model.

### PI3K-mTOR inhibitor and Paclitaxel

**Figure 2:**
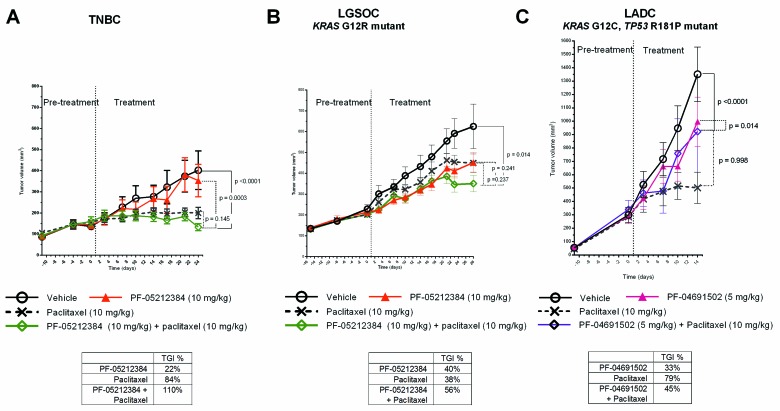
Combined Paclitaxel and PI3K-mTOR inhibitor in PTEN-deficient PDX Tumor growth of **A.** triple-negative breast cancer (TNBC); **B.** *KRAS* mutant (G12R) low-grade ovarian cancer (LGSOC) and **C.** *TP53* (R181P) and *KRAS* (G12C) mutant lung adenocarcinoma (LADC). The TNBC and LGSOC models were treated with vehicle, PF-05212384 (10 mg/kg, twice weekly, intravenously, paclitaxel (10 mg/kg, twice weekly, intraperitoneally) or the combination of both agents. The LADC was treated with vehicle, PF-04691502 (5 mg/kg, daily, oral gavage), paclitaxel (10 mg/kg, twice weekly, intraperitoneally) or the combination of both agents. Relative tumor volumes are displayed as mean +/- SE. p: p value for daily tumor volume change for each arm in comparison to vehicle arm. TGI%: percentages of tumor growth inhibition in comparison to vehicle arm.

Although PF-05212384 and PF-04691502 showed no significant activity as single agents, paclitaxel induced TGI > 50% in the TNBC and LADC models (Table 1). The combination arm only induced TGI > 50% in the LGSOC and TNBC models (Figure 2A, 2B). Although the difference between the paclitaxel single agent and paclitaxel + PF-05212384 arms were not statistically significant, we observed signs of additive activity by adding PF-05212384 to paclitaxel in these two models; In the TNBC model, the combination arm induced tumor regression, which was not achieved in the paclitaxel single agent arm. In the LGSOC model, the combination arm achieved a TGI > 50%, while such TGI was not achieved by any of the single agent arm achieved (Table 1). The results of the LADC model (Figure 2C) should be evaluated with caution, as several mice in the paclitaxel arm suddenly died between weeks 1 and 2 of treatment. These deaths were directly attributed to dose initially selected of paclitaxel (15 mg/kg), as no new events were observed in any of the models once the paclitaxel dose was reduced to 10 mg/kg. No significant weight loss was observed in any of the treatment arms in the three models.

### PI3K-mTOR inhibitor and dacomitinib

**Figure 3:**
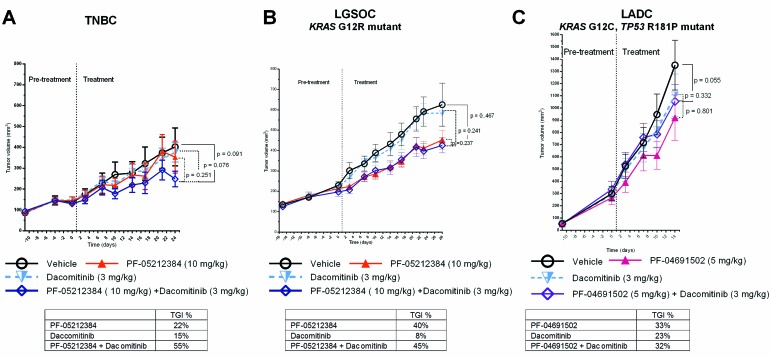
Combined dacomitinib and PI3K-mTOR inhibitor in PTEN-deficient PDX Tumor growth of **A.** triple-negative breast cancer PDX (TNBC), *KRAS* mutant (G12R) low-grade ovarian cancer (LGSOC) and *TP53* (R181P) and *KRAS* (G12C) mutant lung adenocarcinoma (LADC). The TNBC and LGSOC models were treated with vehicle, PF-05212384 (10 mg/kg, twice weekly, intravenously, dacomitinib (3 mg/kg daily, oral gavage) or the combination of both agents. The LADC was treated with vehicle, PF-04691502 (5 mg/kg, daily, oral gavage), dacomitinib (3 mg/kg daily, oral gavage) or the combination of both agents. Relative tumor volumes are displayed as mean +/- SE. p: p value for daily tumor volume change for each arm in comparison to vehicle arm. TGI%: percentages of tumor growth inhibition in comparison to vehicle arm.

As single agents, neither the PI3K-mTOR inhibitors nor dacomitinib achieved a TGI of 50%, (Table 1 and Figure 3). The combination arm only induced a mean TGI > 50% in the TNBC model (TGI = 55 %), but this did not achieve statistical significance according to the mixed effect model (*p* = 0.09) (Table 1). No relevant weight loss or other toxicity was observed in any of the arms.

### Evaluation of downstream effector phosphoproteins

**Figure 4:**
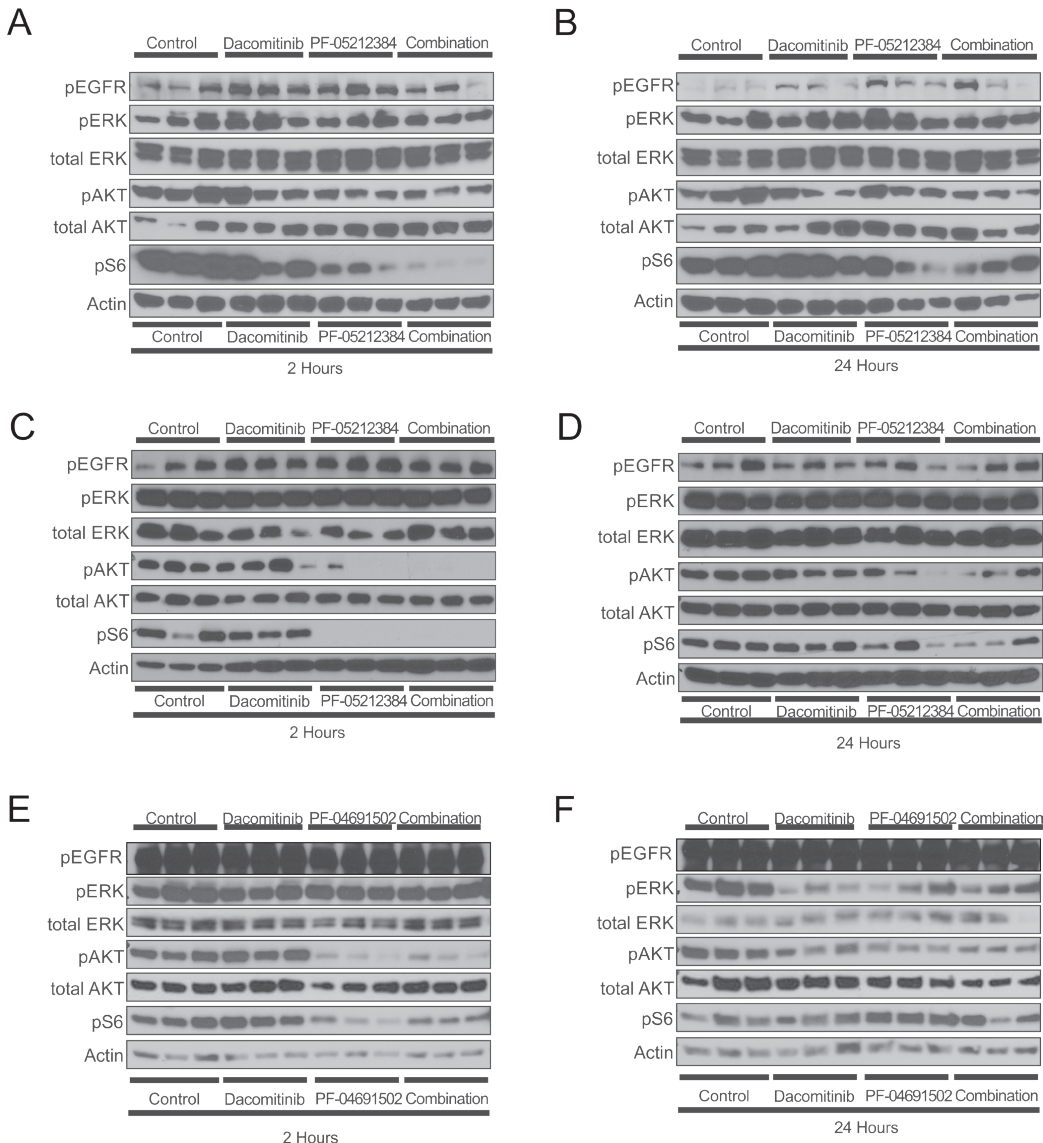
Western blot analysis of the indicated proteins in three independent tumors of the triple-negative breast cancer (TNBC), *KRAS* mutant (G12R) low-grade ovarian cancer (LGSOC) and *TP53* (R181P) and *KRAS* (G12C) mutant lung adenocarcinoma (LADC) The TNBC **A.**, **B.** and LGSOC **C.**, **D.** models were treated with vehicle, PF-05212384 (10 mg/kg, twice weekly, intravenously, dacomitinib (3 mg/kg daily, oral gavage) or the combination of both agents. The LADC model **E.**, **F.** was treated with vehicle, PF-04691502 (5 mg/kg, daily, oral gavage), dacomitinib (3 mg/kg daily, oral gavage) or the combination of both agents. Tumors were collected 1 hour after treatment (A, C, E) or 24 hours after treatment (B, D, F).

Despite the limited anti-tumor effect induced by the PI3K-mTOR inhibitors single agents or in combination with dacomitinib, downstream inhibition of the PI3K/AKT/mTOR pathway was observed in all of the arms containing a PI3K-mTOR inhibitor (Figure 4A-4F): all models exhibited pS6 inhibition and the LGSOC and LADC models also exhibited pAKT inhibition. The inhibition of these phospho-proteins was, in general, greater in the combination arm, which might reflect the requirement for dual inhibition of the tyrosine kinase receptor and PI3K to completely abrogate the pathway. Substantial inhibition of AKT and S6 phosphorylation was observed 2 hours after treatment; while their phosphorylation levels partially recovered by 24 hours. The differences in phosphorylation patterns between the LADC, TNBC and LGSOC models might be partially due to the different molecular backgrounds, but also to the different pharmacokinetic profiles of PF-04691502 and PF-05212384.

Although the addition of dacomitinib was expected to induce some degree of inhibition in the MAPK signaling pathway, no effects on pEGFR, nor on pERK levels was observed in the three models (Figure 4A-4F).

## DISCUSSION

We simultaneously evaluated multiple combinations of novel therapeutic strategies using PDXs as an innovative platform for preclinical evaluation of these combinations. The selected models harbored molecular alterations commonly described in the matched tumor types, such as loss of PTEN expression present in around 30% in patients with TNBC [35] and LADC [36] or *KRAS* mutation described in around one third of LADC [37] and LGSOC [38]. The response to conventional chemotherapy in the three selected PDXs resembled the response in the matching tumor types reported in clinical trials and retrospective series, from a chemo-sensitive model represented by the LADC to a chemo-resistant model represented by the LGSOC [31, 33, 34, 39]. We observed that the addition of PF-05212384 to cisplatin enhanced the antitumor activity of cisplatin suggesting a chemo-sensitizing effect. There is preclinical evidence supporting the role of the PI3K-AKT-mTOR pathway in DNA repair. The beta isoform of PI3K (p110β) seems to have a role sensing DNA damage and facilitating the binding to DNA of DNA repair proteins from the ATM and ATR pathways [40]. Loss of p110β was found to induce genomic instability, whereas 110β inhibition increased sensitivity to DNA damaging agents [40]. The PI3K-mTOR pathway also regulates the expression of certain DNA repair proteins, such as BRCA1 [41] and FANCD2 [3], as decreased levels of these proteins are observed upon PI3K-mTOR inhibition. PTEN also is involved in chromosomal integrity and DNA repair, specifically the fraction of PTEN localized in the nucleus [42, 43]. In xenografts established from PTEN-deficient cell lines, the addition of a PI3K inhibitor increased the antitumor activity of cisplatin [43]. Our experiments did not assess DNA damage markers or DNA repair proteins, so the specific mechanism by which the addition of PF-05212384 might have induced cisplatin chemo-sensitization was not fully clarified. Furthermore, the use of a pan-isoform PI3K-mTOR inhibitor, does not allow to discern whether the chemo-sensitization induced by PF-05212384 depends on an individual PI3K isoform or whether simultaneous inhibition of different PI3K isoforms is needed. Experiments comparing the activity of pan-isoform PI3K inhibitors and novel beta-isoform PI3K inhibitors combined with chemotherapy would help understanding the mechanism of chemo-sensitization observed in our experiments.

The effects of adding a PI3K-mTOR inhibitors to chemotherapy has varied among the three models tested: from a synergistic effect in the TNBC model to lack of effect in the LADC model; whereas an intermediate effect was achieved in the LGSOC model. These differences between these models may be probably related to their variable molecular backgrounds. The LADC had a *TP53* missense mutation causing a complete loss of function of p53 [44]. *TP53* mutations have been identified as potential mechanisms of resistance to PI3K inhibition in cell lines and in patients participating in the phase I clinical trial evaluating the alpha-specific PI3K inhibitor BYL719 [45]. Further research is warranted to characterize the role of *TP53* status on the activity of the PI3K inhibitors. The LADC and LGSOC models also harbored *KRAS* mutations (G12C and G12R respectively). Different *KRAS* mutations seemed to signal preferentially through different downstream pathways [46]; *KRAS* mutations signaling preferentially through the MAPK pathway, such as G12C, are a well-known resistance factor to PI3K inhibition [47]; whereas *KRAS* G12R signals through both the ERK and PI3K pathways, which might explain the moderate chemo-sensitization achieved in the LGSOC, disease characterized by its intrinsic relative chemo-resistance. Although the addition of a PI3K-mTOR inhibitor to chemotherapy might be a strategy to increase the antitumor activity of chemotherapy in LGSOC, the combination of MEK and PI3K inhibitors would probably be a more relevant combination based on the high response rate observed in this patient population in the early clinical trials evaluating these combinations [48, 49].

In the experiments evaluating the combination of PI3K-mTOR inhibitors and dacomitinib, no statistically significant difference was observed between the combination arm and the vehicle arm. This lack of activity contrasts with results from the western-blot, in which target inhibition was observed in the arms containing the PI3K-mTOR inhibitors. No significant pEGFR inhibition was observed in the dacomitinib containing arms which might be related to the dose selected, lower than in prior *in vivo* experiments [27, 28, 50, 51]. The lack of MAPK pathway inhibition in the arm combining PIK3-mTOR inhibitor and dacomitinib may be related to an insufficient pEGFR inhibition or to the presence of activating events in the MAPK pathway, such as *KRAS* mutations, which may constitute potential mechanisms of resistance to this treatment combination.

The preclinical results generated by our study have supported the translation of these combinations into the clinic and helped define the patient populations for each combination. The combination of cisplatin and PF-05212384 in patients with TNBC is currently under evaluation in a phase I clinical trial (NCT01920061), which has three parallel arms evaluating PF-05212384 in combination with cisplatin, docetaxel, and dacomitinib respectively. Although no molecular selection is required, paired biopsies are being collected for biomarker analysis. This trial should provide further insights into the role of PI3K-mTOR inhibitors as chemosensitizing agents and could help to clarify the effect of PF-05212384 on DNA damage and repair and the role of different molecular biomarkers, such as PTEN deficiency, on such effect. The results provide some evidence that preclinical studies of novel targeted drugs in PDX may reveal the treatment response heterogeneity, which could be informative in refining the design of early stage clinical trials for these drugs.

## MATERIALS AND METHODS

### Drugs

The two PI3K-mTOR inhibitors evaluated, PF-05212384 and PF-04691502, were provided by Pfizer Inc. (New York City, NY), as well as the pan-HER inhibitor dacomitinib; cisplatin (Teva Parenteral Medicines, Inc., Irvine, CA) and paclitaxel (Sigma-Aldrich Corporation, St. Louis, MO) were purchased from the hospital pharmacy.

### PDX models

The three PDX models were selected from a repository at the University Health Network (UHN) that has been established with patient consent and in accordance to the guidelines of the UHN Human Research Ethics Board. Animal experiments were performed at the Toronto Centre for Phenogenomics in compliance with regulatory guidelines and a protocol approved by the UHN Animal Care Committee. Three models were selected based on their deficient PTEN expression as assessed by immunohistochemistry: a TNBC model established from a liver metastasis biopsy, a LGSOC model established from an ascites sample, and a lung adenocarcinoma (LADC) model established from a surgical specimen. Breeders were purchased from Jackson Laboratories (Bar Harbor, ME) and were used for up to four generations to avoid genetic drift. Cryobanked tumor fragments were revived and expanded as donors in 2-3 serial mouse generations at the subcutaneous flank site of non-obese diabetic severe combined immunodeficiency (NOD-SCID) NOD.CB17-Prkdc^scid^/J mice.

### PDX therapeutic studies

In the experimental phase, groups of 12 tumor-bearing mice were randomized to treatment or vehicle once tumors reached an average volume of 250 mm^3^ by caliper measurements.

Treatment doses were selected according to previously performed maximum tolerated dose experiments and were refined further based on the observed toxicity profile. Three different drug combinations were evaluated with their matching single agent and vehicle arms. Treatment arm details are summarized in Table 2. In the LADC model, both PI3K-mTOR inhibitors—PF-05212384 and PF-04691502—were tested. However, in the subsequent models, only PF-05212384 was tested as PF-05212384 would be the compound that would be further evaluated in clinical trials based on additional clinical data that became available before starting the TNBC and LGSOC models. Tumor size and mouse weight were evaluated twice per week. Tumor volume was calculated using the formula = length^2^ x width x 0.52. Mice were sacrificed once tumors reached 1500 mm^3^ or when other human endpoints were observed in compliance with regulatory guidelines of the Institutional Animal Care Committee. Pieces of extracted tumors were snap-frozen in liquid nitrogen and formalin-fixed and paraffin-embedded for further analysis.

**Table 2:** Treatment arms, dose, frequency and administration route

Cisplatin + PI3K-mTOR inhibitor experiment				
**Model**	Drug	Dose	Frequency	Route
**LADC 1** **TNBC** **LGSOC**	Cisplatin	3 mg / kg	Weekly	IP
	PF-05212384	10 mg/ Kg	2 times / week	IV
	Cisplatin	3 mg / kg	weekly	IP
	PF-05212384	10 mg/ Kg	2 times / week	IV
	Vehicle		weekly	IP
			2 times / week	+ IV
**Paclitaxel + PI3K-mTOR inhibitor experiment**				
**Model**	Drug	Dose	Frequency	Route
**LADC**	Paclitaxel	15 mg / kg ^2^	2 times / week	IP
	PF-04691502	5 mg / kg	daily	PO
	Paclitaxel	15 mg / kg	2 times / week	IP
	PF-04691502	5 mg / kg	daily	PO
	Vehicle		daily	PO +
			2 times / week	IP
**TNBC** **LGSOC**	Paclitaxel	10 mg / kg	2 times / week	IP
	PF-05212384	10 mg / kg	2 times / week	IV
	Paclitaxel +	10 mg / kg	2 times / week	IP
	PF-05212384	10 mg / Kg	2 times / week	IV
	Vehicle		2 times / week	IP + IV
**Dacomitinib + PI3K-mTOR inhibitor**				
**Model**	Drug	Dose	Frequency	Route
**LADC**	Dacomitinib	3 mg / kg	daily	PO
	PF-04691502	5 mg / kg	daily	PO
	Dacomitinib	3 mg / kg	daily	PO
	PF-04691502	5 mg / kg	daily	PO
	Vehicle		daily	PO
**TNBC** **LGSOC**	Dacomitinib	3 mg / Kg	daily	PO
	PF-05212384	10 mg/ Kg	2 times / week	IV
	Dacomitinib	3 mg / Kg	daily	PO
	PF-05212384	10 mg/ Kg	2 times / week	IV
	Vehicle		daily	PO
			2 times / week	IV

LADC: non-small cell lung cancer; TNBC: triple negative breast cancer; LGSOC: low-grade serous ovarian cancer; IP: intraperitoneal; IV: intravenous; PO: oral gavage; ^1^ In the LADC model, PF-05212384 at a dose of 15 mg / kg was initially tested. Several sudden deaths occur after the first drug administration; thus, PF-05212384 was reduced to 10 mg / kg in subsequent administrations. The models evaluated afterwards (TNBC and LGOSC) received a dose of 10 mg / kg. ^2^ Paclitaxel was initially evaluated at a dose of 15 mg / kg in the LADC and subsequently changed to 10 mg/kg based on several unexpected deaths in the paclitaxel-containing arms. Paclitaxel was tested at a dose of 10 mg / kg in the remaining models.

A parallel acute dose experiment was performed to evaluate the pharmacodynamic effect of PF-05212384, PF-04691502, and dacomitinib on downstream effectors in the treatment groups. Three mice replicates were harvested at 2 and 24 hours post-dosing.

### Genomic characterization of patient-derived tumor xenografts

PDXs in the repository were characterized using the OncoCarta Panel v 1.0 (238 mutations in 19 key oncogenes) on the MassARRAY System (Agena Bioscience, San Diego, CA) to determine their tumor mutational profile.

### Immunoblots of downstream effector proteins

Aliquots of approximately 50 mg of tissue were mixed with 1 ml of lysis RIPA buffer (R 0278, Sigma, Saint Louis, MO) buffer containing a protease inhibitor cocktail (11 836 170 001, Roche Diagnostics GmbH, Mannheim, Germany), 8 mM sodium orthovanadate, and 0.2 mM phenylmethanesulfonyl fluoride. Protein extracts were collected after tissues were homogenized (1 min) and centrifuged (20000 × g, 20 min). Aliquots of 30 µg of protein were mixed with an equal volume of 2 X SDS-PAGE sample loading buffer, and resolved by SDS-PAGE. Resolved samples were transferred to polyvinylidene fluoride (PVDF) membranes by using Trans-Blot^®^ Turbo™ Midi PVDF Transfer Packs, 170-4157 BioRad, Hercules, CA). Membranes were blocked with 5% nonfat dry milk in Tris-Buffered Saline with Tween 20 (1X TBST). Blots were incubated overnight at 4°C with primary antibodies (EGFR Tyr 1068, ERK, pERK1/2 Thr202/Tyr204, AKT, pAKT Ser 473, pS6 Ser235/236, and β-actin) at 1:1000 dilutions, followed by incubation with a 1: 4000 dilution of HRP-linked anti-rabbit IgG secondary antibodies for one hour . All antibodies used for blotting were from Cell Signaling Technology, Danvers, MA. Immunoreactive protein bands were detected by ECL-Prime Western blotting detection reagent (RPN2236, GE Healthcare, Little Chalfont, UK).

### Immunohistochemistry

PTEN immunohistochemistry was performed using the Ventana Benchmark XT autostainer for anti-PTEN (138G6, Cell Signaling Technology, Danvers, MA) with the iVIEW DAB detection system (Ventana Medical Systems, Inc., Tucson, AZ). Absence of staining of tumor cell cytoplasm was classified as loss of expression whereas faint staining was considered as low PTEN expression.

### Statistical analysis

Mixed effect model was used to test the differences in tumor growth rates overtime between treatment and control groups within each PDX model. SAS 9.2 (SAS Institute Inc., Cary, NC) was used for the analysis.

Relative mean tumor growth was calculated with the formula

$$\frac{\Delta \;\text{mean tumor volume treatment arm}-\Delta \;\text{mean tumor volume control arm}}{\Delta \;\text{mean tumor volume treatment arm}}\times 100$$

Tumor volume were plotted as mean +/- standard deviation with GraphPad Prism 6 (GraphPad Software Inc., San Diego, CA).

## SUPPLEMENTARY MATERIALS FIGURE


