# Supplementary material for: Novel combinations of PI3K-mTOR inhibitors with dacomitinib or chemotherapy in PTEN-deficient patient-derived tumor xenografts
Source: Oncotarget. 2017 Jul 8;8(49):84659–70. doi: 10.18632/oncotarget.19109 (PMC5689564; doi:10.18632/oncotarget.19109)
Supplement: Supplementary file 1 [file oncotarget-08-84659-s001.pdf]

Novel combinations of PI3K-mTOR inhibitors with dacomitinib or chemotherapy in PTEN-deficient patient-derived tumor xenografts

SUPPLEMENTARY MATERIALS

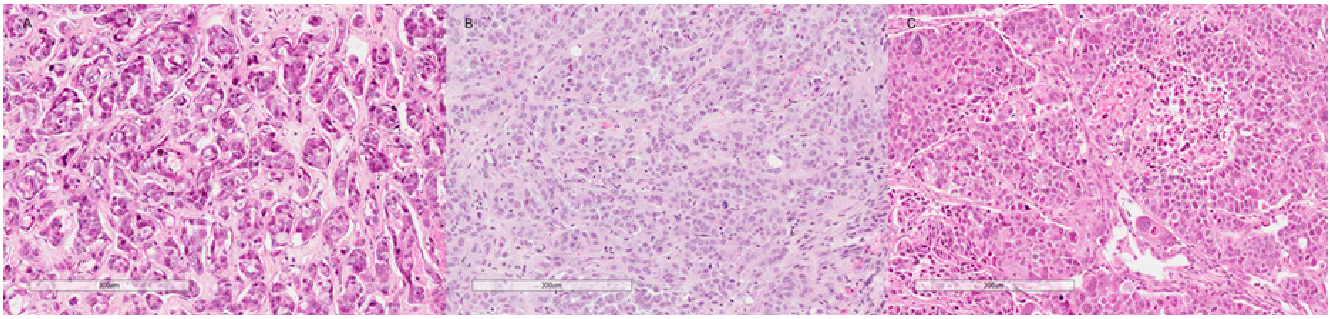

Supplementary Figure 1: Hematoxylin and eosin stain of patient derived xenografts: A) Triple Negative Breast Cancer model; B) Low Grade Serous Ovaria Cancer model; C) Lung Adenocarcinoma model
